# Tracing Genetic Divergence and Phylogeographic Patterns of *Gekko gecko* Linnaeus, 1758 (Squamata: Gekkonidae) Across Southeast Asia Using *RAG1* Sequence

**DOI:** 10.3390/ani15203004

**Published:** 2025-10-16

**Authors:** Panida Laotongsan, Warayutt Pilap, Chavanut Jaroenchaiwattanachote, Pattana Pasorn, Jatupon Saijuntha, Wittaya Tawong, Watee Kongbuntad, Komgrit Wongpakam, Khamla Inkhavilay, Mak Sithirith, Chairat Tantrawatpan, Weerachai Saijuntha

**Affiliations:** 1 Walai Rukhavej Botanical Research Institute, Mahasarakham University, Maha Sarakham 44150, Thailand; panida_walai@yahoo.com (P.L.); warayutt@msu.ac.th (W.P.); pattanapasorn@gmail.com (P.P.); komwongpa@gmail.com (K.W.); 2 Center of Excellence in Biodiversity Research, Mahasarakham University, Maha Sarakham 44150, Thailand; chavanut.j@msu.ac.th; 3 Faculty of Engineering, Mahasarakham University, Maha Sarakham 44150, Thailand; jatupons2534@gmail.com; 4 Center of Excellence in Biodiversity, and Center of Excellence in Research for Agricultural Biotechnology, Naresuan University, Phitsanulok 65000, Thailand; wittayat@nu.ac.th; 5 Department of Agricultural Sciences, Faculty of Agriculture Natural Resources and Environment, Naresuan University, Phitsanulok 65000, Thailand; 6 Program in Biotechnology, Faculty of Science, Maejo University, Chiang Mai 50290, Thailand; watee@mju.ac.th; 7 Center of Excellence in Biodiversity, National University of Laos, Vientiane 7322, Laos; khamla.inkhavilay@nuol.edu.la; 8 Cambodia Development Resource Institute (CDRI) Tuol Kork, Phnom Penh 120508, Cambodia; maksithirith@gmail.com; 9 Division of Cell Biology, Department of Preclinical Sciences, Faculty of Medicine, and Center of Excellence in Stem Cell Research and Innovation, Thammasat University, Rangsit Campus, Pathum Thani 12120, Thailand; 10 Biomedical Science Research Unit, Faculty of Medicine, Mahasarakham University, Maha Sarakham 44000, Thailand

**Keywords:** tokay gecko, genetic variation, genetic structure, phylogeny, haplotype network, species delimitation

## Abstract

**Simple Summary:**

The tokay gecko (*Gekko gecko*) is a colorful lizard found in Southeast Asia and is widely used in traditional medicine. In this study, we analyzed DNA from tokay geckos in Thailand, Laos, and Cambodia to understand their genetic differences. We found clear genetic variation between populations and discovered three main genetic lineages. These results suggest that what is thought to be one species may include hidden, distinct groups. This information is important for protecting the species and managing its trade.

**Abstract:**

The tokay gecko (*Gekko gecko*) is a widely distributed lizard species in Southeast Asia, with significant importance in traditional medicine and the pet trade. Previous studies using mitochondrial DNA sequences revealed extensive genetic variation across its range, indicating the presence of distinct evolutionary lineages. In this study, we assessed the nuclear genetic variation and phylogenetic pattern of *G. gecko* using the recombination activating gene 1 (*RAG1*). We analyzed 105 *RAG1* sequences from 16 localities across Thailand, Laos, and Cambodia, along with additional sequences from GenBank. Sequence analysis revealed 20 variable sites and 20 haplotypes (TgR1–TgR20). Haplotype network and phylogenetic analyses revealed strong regional structuring and at least three distinct evolutionary lineages (A–C), supported by the species delimitation test (PTP). Both red- and black-spotted morphs were present in different clades, indicating that external coloration does not correspond to genetic differentiation at this locus. Our results support the presence of distinct evolutionary lineages in *G. gecko* and emphasize the importance of integrative taxonomy for accurate species delimitation. These findings have implications for conservation, sustainable management, and regulation of international trade in this commercially exploited species.

## 1. Introduction

The tokay gecko (*Gekko gecko*), a large nocturnal lizard species in the family Gekkonidae, is widely distributed across tropical and subtropical Asia, including much of Southeast Asia. Additionally, it has been introduced to several regions outside its native range, such as the United States, Mexico, Puerto Rico, Martinique, and Hong Kong [1]. Beyond its ecological role, *G. gecko* is highly valued in traditional medicine across Asia, where it is believed to possess therapeutic properties against asthma, tuberculosis, cancer, diabetes, skin disorders, and even HIV/AIDS [2]. Demand is intense: over 1.2 million dried individuals are exported annually from Java [3], and an estimated 2–5 million individuals are exported from Thailand each year [4]. Additional sourcing occurs in Cambodia and Laos [5,6], with most trade directed toward China, Hong Kong, and Malaysia for use in traditional Chinese medicine [7].

Two main color morphs of *G. gecko* are recognized, namely the black-spotted form, occurring mainly in Guangxi and Yunnan, southern China, and the red-spotted form, which is widespread across Asian countries [8]. Morphological, behavioral, and genetic differences between these two morphs have been reported, including differences in coloration [9], vocalizations [10], karyotypes [8], RAPD profiles [11], allozymes [5], and both nuclear and mitochondrial DNA markers [6,9,12,13].

In mainland Southeast Asia, studies using multi-locus enzyme electrophoresis and mitochondrial DNA sequences have demonstrated considerable intra- and inter-population diversity, with evidence for multiple genetic clades [5,6]. For example, six haplogroups (G1–G6) from various localities in Southeast Asia have been identified based on mitochondrial tRNA-Gln/tRNA-Met/partial ND2 sequence, which were grouped into five clades (A–E) [6]. Genetic breaks corresponded to major mountain ranges (e.g., Phetchabun, Dong Paya Yen, Phi Pan Nam) and river barriers (e.g., Mekong), suggesting that geographic barriers strongly influence lineage divergence [5,6]. These findings demonstrate distinct evolutionary lineages and possible species-level distinctions, underscoring the need for integrative taxonomy.

While mitochondrial DNA provides high resolution for detecting recent divergence, it represents only the maternal lineage. Nuclear markers provide complementary biparental inheritance and are critical for resolving deeper evolutionary history. The recombination activating gene 1 (*RAG1*) is a single-copy nuclear coding gene, which encodes an essential enzyme for initiating V(D)J (variable, diversity, and joining gene segments) recombination during lymphocyte development. It has been proven useful in vertebrate phylogeny and in exploring population structure and divergence in fishes and reptiles due to its moderate mutation rate, low paralogy, biparental inheritance, and conserved coding regions [14]. Previous studies have successfully applied *RAG1* in reptiles, including *G. gecko* [1,12].

In this study, we analyze *RAG1* sequences from *G. gecko* across Thailand, Lao PDR, and Cambodia, together with additional GenBank records, to investigate genetic divergence, population structure, and phylogenetic pattern. Specifically, we test for phylogeographic structuring, evaluate congruence with mitochondrial studies, and assess potential cryptic species using multiple delimitation methods. These findings aim to clarify the evolutionary history of *G. gecko* and provide a framework for conservation and sustainable trade management.

## 2. Materials and Methods

### 2.1. Sample Collection

**Figure 1:**
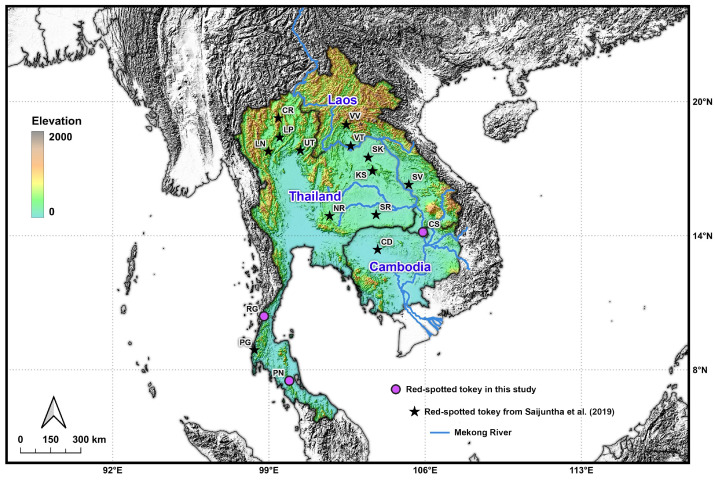
Sampling localities of *Gekko gecko* populations across Thailand, Laos, and Cambodia. Each locality code corresponds to those listed in Table 1. Purple circles indicate sampling sites from the present study, while dark stars represent localities from a previous study [6].

Tokay gecko samples from 13 localities in Thailand, Laos, and Cambodia were obtained from a previous study [6]. An additional 23 samples were newly collected from three localities in Thailand and Laos (Table 1 and Figure 1). Geckos were captured using the fishing pole method [15]. Prior to tissue collection, individuals were anesthetized with isoflurane [16] to minimize stress and pain. Each gecko was placed in an induction chamber containing a cotton pad soaked with 3–5% isoflurane. Anesthesia was confirmed by the absence of the righting reflex and reduced response to tactile stimuli. A tail-tip sample (~1–2 mm) was excised aseptically and preserved in 80% ethanol. After recovery, all geckos were released back into their natural habitats. Samples were transported to the Walai Rukhavej Botanical Research Institute, Mahasarakham University, and stored at room temperature until DNA extraction.

### 2.2. DNA Extraction and PCR Amplification

Genomic DNA was extracted from tail tissue using the E.Z.N.A.^®^ Tissue DNA kit (Omega bio-tek, Norcross, GA, USA) following the manufacturer’s protocol. An estimated 700 bp fragment of *RAG1* gene was amplified using primers RAG1-F (5′-CCA GAG GAA GTT CAG CAG TGT C-3′) and RAG1-R (5′-GCT TCC AAC TCA TCA GCT TGT C-3′) [12]. PCR conditions were: 95 °C for 3 min, followed by 35 cycles of 94 °C for 35 s, 57 °C for 45 s, and 72 °C for 90 s, with a final extension step at 72 °C for 5 min. Amplicons were purified with an E.Z.N.A.^®^ Gel Purification Kit (Omega bio-tek, Norcross, GA, USA). The purified PCR products were sent for DNA sequencing at ATGC Co; Ltd., (Pathum Thani, Thailand).

### 2.3. DNA Sequence Analysis

All 553 bp *RAG1* sequences generated in this study were aligned using the ClustalW program version 2.0 [17], and the variable sites between haplotypes were compared in the BioEdit program version 7.2.5 [18]. Molecular diversity indices and haplotype data analysis were calculated using the DnaSp program version 5 [19]. The genetic difference between populations was calculated based on p-distance and Kimura 2-parameter (K2P) distance [20] using the program MEGA version 11 [21]. A minimum-spanning haplotype network was constructed in the PopART version 1.7 (https://popart.maths.otago.ac.nz/, accessed on 30 September 2025) based on a median-joining network [22] using all sequences generated in this study. Analysis of Molecular Variance (AMOVA) was conducted using the Arlequin program version 3.5.2.2 [23]. Furthermore, to investigate the genetic structure across geographical barriers within the study area in this study (Thailand, Laos, and Cambodia), a Spatial Analysis of Molecular Variance (SAMOVA), to cluster the *RAG1* sequences into genetically and geographically homogeneous groups, was conducted in SAMOVA version 1.0 [24]. The analyses were performed for K = 2–8 with 1000 simulated annealing steps from 100 random starting conditions. Due to incomplete coordinate information, the *RAG1* sequences of *G. gecko* from NCBI GenBank were excluded.

### 2.4. Phylogenetic Tree Analysis

Phylogenetic relationships among *G. gecko* were constructed using Bayesian inference (BI), Maximum likelihood (ML), and Neighbor joining (NJ) methods based on *RAG1* sequences. The best-fit evolutionary model (HKY + G) was identified under the corrected Akaike information criterion (AIC) using MrModeltest 2.4 [25]. For Bayesian Inference (BI), the nucleotide substitution model was set in Mesquite v.4.01 [26] with nst = 2 and rates = gamma. The analysis was then performed in MrBayes v.3.2.7 [27] using two parallel runs of four Markov chains for 30 million generations, sampling every 1000 generations. Convergence was assessed in Tracer 1.7.2 [28], with the first 25% of trees were discarded as burn-in. Posterior probabilities were estimated from a 50% majority-rule consensus tree. ML and NJ analyses were conducted in MEGA XI [21] with 1000 bootstrap replicates, using the HKY + G model and Kimura 2-parameter (K2P), respectively. To provide phylogenetic context, representative sequences from related gecko taxa (*Cyrtodactylus*, *Dravidogecko*, *Agaura*, *Tropiocolotes*, and *Hemidactylus*) were included, with *Cnemaspis tucdupensis* designated as the outgroup.

### 2.5. Species Delimitation Analysis

Species boundaries were evaluated using three single-locus species delimitation methods in this study on *G. gecko RAG1* sequences. Automatic Barcode Gap Discovery (ABGD) [29], Assemble Species by Automatic Partitioning (ASAP) [30], and the tree-based Poisson tree (PTP) [31]. The PTP method was applied to *RAG1*-based phylogeny using the web server (https://mptp.h-its.org/#/tree/; accessed on 28 July 2025). ABGD and ASAP analyses were implemented in the bioinformatic toolkit iTaxoTools v0.1 [32]. For ABGD, the Kimura (K80) substitution model was employed, with the default maximum and minimum intraspecific distances (Pmax = 0.1, Pmin = 0.001). The barcode gap width was set to 1.5. A non-recursive partition was selected, with a prior maximum intraspecific divergence (*p*) = 2.8 × 10^−3^. For ASAP, the partition with the lowest ASAP score and an appropriate threshold distance (dT) was chosen under the Kimura (K80) model, using a default transition/transversion ratio of 2.0 and a probability of 0.01.

## 3. Results

### 3.1. Phylogenetic Tree and Species Delimitation

**Figure 2:**
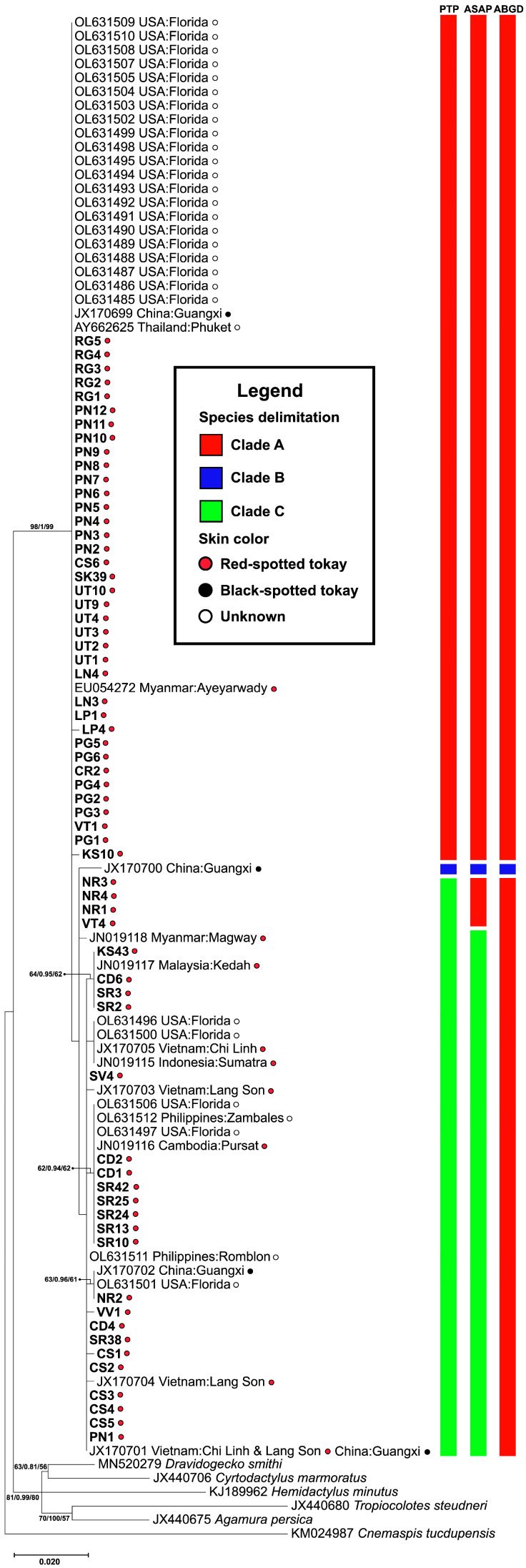
Phylogenetic tree constructed from *RAG1* sequences of *Gekko gecko*. Bootstrap values for Maximum Likelihood (ML) and Bayesian Inference (BI), as well as posterior probabilities for Neighbor Joining (NJ), are indicated above or near the branches. The scale bar represents 0.02 substitutions per nucleotide position. Each bar beside the phylogenetic tree represents a species delimitation method tested by the PTP, ASAP, and ABGD, respectively. The red, blue, and green bars represent clades A, B, and C, respectively.

Phylogenetic analyses based on *RAG1* sequences consistently recovered three well-supported clades (A–C) with species boundaries further evaluated using the Poisson Tree Processes (PTP) method (Figure 2). Clade A contained the majority of individuals, including the samples from Thailand, Myanmar, China, and introduced populations in the USA. Clade B consists of a sample of black-spotted from China and represents a highly supported, genetically distinct group. Clade C comprises individual samples from Thailand, Vietnam, China, Malaysia, and the USA. Clades A and C include both red- and black-spotted tokay geckos, suggesting that coloration alone does not reflect deep genetic divergence.

The genetic distance-based species delimitation analyses using ABGD and ASAP revealed a non-recursive partition of two genetic clades and a recursive partition of three genetic clades, respectively. The barcode gap analysis from both ABGD and ASAP methods indicated multiple narrow intervals within the range of approximately 0.01–0.00%. In ABGD, partitions 1 and 2, with prior maximum intraspecific divergence of *p* = 1.0 × 10^−3^ and *p* = 1.7 × 10^−3^, respectively, produced over-splitting results with 20 genetic groups. Therefore, partition 3 (*p* = 2.80 × 10^−3^), which yielded two genetic groups, was considered the most appropriate for further interpretation. Unlike ABGD, which relies on manually defined prior *P*, ASAP ranks partitions using a scoring system. The best partition, with the lowest ASAP score (ASAP score = 2.00; dT = 0.0036), identified three genetic groups. However, this result was not fully consistent with the phylogenetic tree analyses (ML, NJ, and BI) (Figure 2). In contrast, the tree-based species delimitation method (PTP) inferred three genetic clades based on branching rates, and its partition pattern was the most congruent with phylogenetic analysis. Therefore, the PTP-inferred clades were the putative species boundaries for subsequent analyses.

### 3.2. Haplotype Network

**Figure 3:**
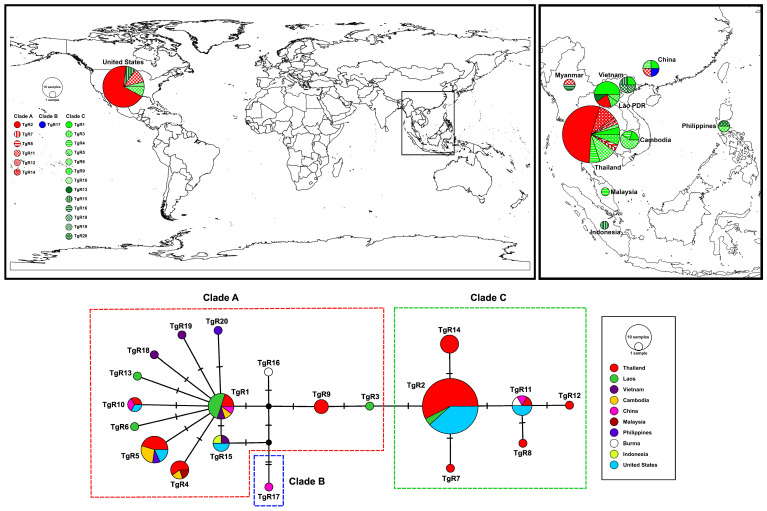
Map showing the frequency distribution of haplotypes from clades A–C across the studied areas (**top panel**) and a neighbor-joining haplotype network constructed from *RAG1* haplotypes of *Gekko gecko* (**bottom panel**). Each color and pattern in the top panel represents distinct clades (A–C) and haplotypes (TgR1–TgR20), respectively, while colors in the bottom panel correspond to different countries. The size of each circle is proportional to the number of individuals sharing that haplotype. Hatch marks on the branches indicate the number of mutational steps between haplotypes.

A total of 20 haplotypes (TgR1–TgR20) were identified from the *RAG1* sequences based on 20 variable nucleotide sites. The median-joining haplotype network revealed three major haplogroups (Figure 3), which corresponded to the species delimitation (PTP) analysis (Figure 2). Haplotypes TgR1 and TgR2 represented central, widely distributed haplotypes. Among them, TgR1 was the most prevalent and geographically widespread, occurring in samples from Laos, Thailand (north, northeastern, and south), and Cambodia. Several other haplotypes (e.g., TgR4, TgR5, TgR6, TgR10, TgR13, TgR15, TgR18–TgR20) radiated from TgR1, indicating localized mutations or population differentiation. Conversely, TgR2 was predominantly composed of sequences retrieved from GenBank, particularly from the USA, Indonesia, and Thailand (Phuket), along with contributions from other Southeast Asian countries (e.g., Vietnam, Philippines, and Malaysia). Several unique haplotypes (e.g., TgR7 and TgR14) were also derived from TgR2, suggesting a divergent clade from that found in mainland Southeast Asia. Notably, TgR11 was shared among Thailand (northeastern), Vietnam, and the Philippines, indicating potential genetic connectivity among these regions. In contrast, TgR17 formed a separate and distinct clade (Clade B).

### 3.3. Spatial Analysis of Molecular Variance (SAMOVA)

The SAMOVA analysis based on *RAG1* data revealed clear genetic structuring of *G. gecko* across mainland and island populations. The proportion of variation among groups (*F*_CT_) was relatively high, ranging from 0.69525 at K = 2 to 0.75897 at K = 8 (Table S2), and all *F*-statistics for each independent run were significant (*p* < 0.01). Although the maximum *F*_CT_ occurred at K = 8, this value indicated possible over-fragmentation. Instead, genetic structure reached an optimal plateau at K = 5 (*F*_CT_ = 0.73889, *p* < 0.001), where the variation among populations within groups (*F*_SC_) sharply declined (*F*_SC_ = 0.00115, *p* < 0.001) before turning negative at K = 6 (*F*_SC_ = −0.06172, *p* < 0.001) (Table S2).

**Figure 4:**
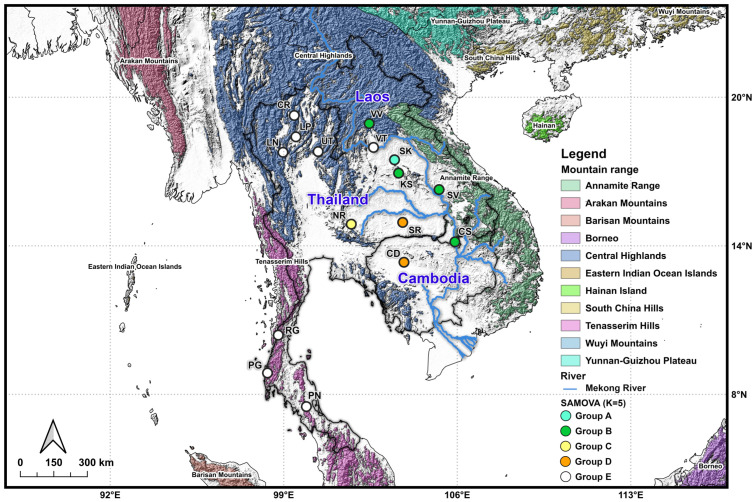
Clustering of genetically and spatially homogenous populations among 16 *G. gecko* populations from Thailand, Laos, and Cambodia using spatial analysis of molecular variance (SAMOVA) at K = 5. Each color on the surface indicated the mountain range, while each different color in the circle represented groups.

At K = 5, the analysis identified five geographically coherent genetic clusters (Figure 4). Group A was confined to SK in northeastern Thailand, corresponding to the Central Highlands. Group B encompassed populations from Laos (VV, SV, and CS) and northeastern Thailand (KS), corresponding mainly to the Central Highlands and Annamite Range. Group C was restricted to NR in northeastern Thailand, while Group D comprised populations from Cambodia (CS) and northeastern Thailand (SR). Finally, Group E represented the isolated population from Laos (VT), northern Thailand (CR, LP, LN, and UT), and southern Thailand (RG, PG, and PN). The distribution of these groups broadly matched major physiographic regions, with mountain ranges and river systems (e.g., the Mekong River) serving as likely barriers to gene flow.

### 3.4. RAG1 Sequence Variation

A total of 105 *RAG1* sequences, each with a length of 553 bp, were analyzed across 16 geographical localities examined in this study and deposited in GenBank under accession numbers PX432629–PX432691, along with 6 additional localities retrieved from GenBank. Sequence comparison revealed 20 variable nucleotide sites (3.6%), with 13 variable amino acid positions after translation (Table S1). The number of segregating sites (S) varied from 5 to 13. The number of haplotypes (h) ranged from 6 to 13 among populations, with several populations exhibiting unique haplotypes. Haplotype diversity (Hd) ranged from 0.396 ± 0.075 to 0.865 ± 0.030, with an average of 0.772 ± 0.039, and nucleotide diversity (π) values ranged from 0.0009 ± 0.0002 to 0.0030 ± 0.0003, with an average of 0.0049 ± 0.0003 (Table 2).

### 3.5. Genetic Differences and Genetic Structure

Pairwise genetic distances among *G. gecko* populations from the three genetic clades (A–C) were calculated using both the Kimura 2-Parameter (K2P) model (lower triangle) and uncorrected *p*-distance (upper triangle) based on *RAG1* sequences (Table 3). The lowest genetic distances were observed between clade C and the other clades (*p*-distance = 0.0082; K2P = 0.0082–0.0083), whereas the highest genetic distances were found between clade A and clade B (*p*-distance = 0.0110; K2P = 0.0111).

Analysis of molecular variance (AMOVA) based on *RAG1* sequences revealed pronounced genetic structuring among *G. gecko* populations across different geographic scales (Table 4). When populations were grouped into different countries, namely Thailand, Laos, Cambodia, and USA, 5.26% of the variation was found among groups (*F*_CT_ = 0.05257, *p* > 0.05), 56.39% among populations within groups (*F*_SC_ = 0.53569, *p* < 0.01), and 48.87% within populations, resulting in an overall *F*_ST_ of 0.51128 (*p* < 0.01). When populations were grouped according to the SAMOVA-defined clusters (Groups A–E), the proportion of genetic variation among groups increased substantially to 73.89%, with a high and significant fixation index (*F*_CT_ = 0.73889, *p* < 0.01), suggesting strong genetic structuring among these groups. In contrast, only 0.03% of the variation occurred among populations within groups, as indicated by a low fixation index (*F*_SC_ = 0.00115, *p* < 0.01), while 26.08% of the variation was found within populations (*F*_ST_ = 0.73919, *p* < 0.01). These results demonstrate strong genetic structuring and restricted gene flow among the SAMOVA-defined groups of *G. gecko*, highlighting substantial genetic divergence among geographically separated populations.

## 4. Discussion

This study reveals significant nuclear genetic divergence and population structure in *G. gecko* across Southeast Asia using the *RAG1* gene. The previous studies suggest the presence of multiple evolutionary lineages and possible cryptic species of G. *gecko* in Southeast Asia [5,6]. These findings complement and extend earlier mitochondrial studies that uncovered deep population structure and highlighted the influence of geographic barriers in shaping genetic diversity [6]. The mitochondrial *ND2* study by Saijuntha et al. [6] analyzed 166 red-spotted tokay geckos from 23 localities and identified six major haplogroups (G1–G6), which were associated with five clades (A–E). Strong phylogeographic structuring was observed, with genetic breaks corresponding to mountain ranges such as Phetchabun, Dong Paya Yen, and Phi Pan Nam, and rivers like the Mekong. These natural features appear to have restricted gene flow, leading to regional lineage divergence. Our nuclear *RAG1* results parallel these findings, revealing high genetic differences, especially among Thailand, Laos, and Cambodia, and identifying distinct clades with limited haplotype sharing.

While mitochondrial markers such as *ND2* and *Cyt-b* often provide higher resolution for detecting recent divergence, they are maternally inherited and may not reflect the full evolutionary history of a species [6,9,12,13]. Nuclear markers like *RAG1* provide independent biparental information and are critical for resolving deeper phylogenetic relationships and confirming species boundaries [1,12]. In our study, the combination of *RAG1* haplotype network, phylogenetic tree, and species delimitation (PTP) analyses confirmed regional divergence and supported the presence of three major *RAG1* clades, partially corresponding to the mitochondrial clades found in previous studies. Additional support for this pattern comes from Fieldsend et al. [1], who reported two distinct mito-nuclear lineages in *G. gecko* populations introduced to Florida, matching lineages from the native range. These lineages showed reproductive isolation, suggesting they may represent separate species. Likewise, Wang et al. [12] and others have noted consistent nuclear-mitochondrial differentiation between red- and black-spotted morphotypes, adding weight to the hypothesis of cryptic speciation.

However, in this study, the *RAG1* gene did not clearly distinguish between the black- and red-spotted morphotypes of *G. gecko*, suggesting low genetic differentiation at this nuclear locus between these color forms. The black-spotted form is generally smaller and darker, whereas the red-spotted form is larger with red markings. Although morphological, karyotypic, and genetic differences have led some studies to propose their recognition as separate species, their taxonomic status remains controversial [33]. This suggests that the observed color polymorphism may not be strongly associated with genetic divergence but may instead reflect phenotypic plasticity, recent divergence, ongoing gene flow, or selection related to ecological conditions. Previous research has also indicated that evolutionary transitions to color polymorphism are often associated with increased diversification rates in lizards [34]. Furthermore, a recent study showed that the black- and red-spotted forms of *G. gecko* occupy distinct ecological niches, with niche differentiation primarily influenced by climatic factors such as isothermality, temperature seasonality, and precipitation during the warmest quarter. These results suggest that ecological divergence, together with morphological variation, may contribute to maintaining their separate distributions and potential taxonomic distinction [33].

Nuclear markers such as *RAG1*, which evolve more slowly than mitochondrial genes, often lack resolution for detecting recent or shallow divergences, particularly within morphologically variable but genetically cohesive lineages. Interestingly, the presence of a single black-spotted individual from China [12] assigned to clade B, distinct from other sampled individuals, may indicate localized differentiation, introgression from a divergent lineage, or even a cryptic species. This unique placement suggests that while most *RAG1* sequences are highly conserved across morphotypes, some degree of regional or population-specific divergence may exist. However, limited sample sizes for both black- and red-spotted *G. gecko* in certain populations reduce the resolution of our molecular analyses. Consequently, these results should be interpreted with caution, and expanded sampling across broader geographic ranges is needed to better assess genetic differentiation and the potential influence of color polymorphism on population structure.

Furthermore, the tokay gecko’s heavy exploitation for traditional medicine and the pet trade underscores the importance of understanding its genetic structure. Millions of individuals are harvested annually from Southeast Asia [3], raising concerns about the loss of unique genetic lineages. If *G. gecko* comprises a complex of cryptic species, unregulated harvest may disproportionately impact certain lineages and drive local extinctions. Moreover, international trade regulations, including CITES listings, may need to consider lineage-level distinctions for effective protection. In light of these findings, we recommend that future studies use multi-locus nuclear markers, broader geographic sampling (particularly in peninsular Thailand and Malaysia), and morphological/ecological comparisons to clarify species limits. Conservation strategies should also consider the presence of genetically distinct populations when developing region-specific management plans.

## 5. Conclusions

This study highlights significant genetic divergence and population structuring of *G. gecko* across Southeast Asia based on nuclear *RAG1* sequence analysis. The identification of three major genetic lineages, supported by robust statistical approaches, such as AMOVA, SAMOVA, phylogenetic tree, and species delimitation analyses, points to distinct evolutionary lineages within this widely distributed species. Our findings underscore the need for an integrative taxonomic approach combining nuclear and mitochondrial markers, morphology, and ecological data to accurately assess species boundaries. These results contribute valuable information for conservation planning and regulatory frameworks, particularly in light of ongoing habitat loss and intense commercial harvesting of *G. gecko*.

## Figures and Tables

**Table 1:** Sampling localities and other related details for *Gekko gecko* populations collected in Southeast Asia.

Code	Province	Region	Country	Clade (Haplogroup) *	*N*	Coordinate
SV	Savannakhet	Central	Laos	D (G4)	1	16.272846° N/105.266552° E
VT	Vientiane	North	Laos	B (G1)	2	17.975828° N/102.621974° E
VV	Vang Viang (Laos)	North	Laos	B (G1)	1	18.938490° N/102.448177° E
CS **	Champasak	South	Laos	n/a	6	13.954472° N/105.933777° E
LN	Lamphun	North	Thailand	E (G5)	2	17.779125° N/98.970251° E
CR	Chiang Rai	North	Thailand	E (G5)	1	19.265866° N/99.424861° E
UT	Uttaradit	North	Thailand	B (G2)	6	17.809095° N/100.388379° E
LP	Lampang	North	Thailand	E (G5)	2	18.403844° N/99.491655° E
KS	Kalasin	Northeast	Thailand	B (G1)	2	16.936796° N/103.632850° E
NR	Nakhon Ratchasima	Northeast	Thailand	B (G1)	4	14.876044° N/101.724654° E
SK	Sakon Nakhon	Northeast	Thailand	B (G1)	1	17.475612° N/103.469636° E
SR	Surin	Northeast	Thailand	A (G3)	8	14.947288° N/103.799448° E
RG **	Ranong	South	Thailand	n/a	5	10.395722° N/98.779416° E
PG	Phang-nga	South	Thailand	B (G6)	6	8.866375° N/98.345836° E
PN **	Pattalung	South	Thailand	n/a	12	7.508305° N/99.913472° E
CD	Siem Reap	Cambodia	Cambodia	A (G3)	5	13.338748° N/103.850614° E
				Total	64	

* DNA samples and genetic groups (clades and haplogroups) follow Saijuntha et al. [6]. ** Localities of samples collected in this study; n/a = not analyzed.

**Table 2:** Molecular diversity indices of *Gekko gecko* from clades A and C based on PTP species delimitation analysis of *RAG1* sequence.

Populations	Molecular Diversity Indices					
	*n*	S	H	Uh	Hd ± SD	π ± SD
Clade A	61	5	6	6	0.396 ± 0.075	0.0009 ± 0.0002
Clade C	43 *	13	13	13	0.865 ± 0.030	0.0030 ± 0.0003
Total	104 *	18	19	19	0.772 ± 0.039	0.0049 ± 0.0003

*n*, sample size; S, segregation site; H, number of haplotypes; Uh, unique haplotype; Hd, haplotype diversity; π, nucleotide diversity; SD, standard deviation; * black-spotted from China and red-spotted form Vietnam are combined. Locality codes are provided in Table 1. Clade B was excluded because it contained only a single sample.

**Table 3:** Genetic differences based on the *RAG1* gene among different clades of *Gekko gecko*, based on PTP species delimitation analysis, showing Kimura 2-parameter (lower triangle) and *p*-distances (upper triangle).

Clade	A	B	C
A	–	0.0110	0.0082
B	0.0111	–	0.0082
C	0.0082	0.0083	–

**Table 4:** Analysis of Molecular Variance (AMOVA) and spatial AMOVA (SAMOVA) based on *RAG1* sequences of *Gekko gecko* from different geographical localities, with groups defined by different countries.

Source of Variation	d.f.	Ss	Vc	%Va	Fi
**Thailand, Laos, Cambodia, USA**					
Among groups	3	19.608	0.06634	5.26	*F*_CT_ = 0.05257
Among populations within groups	14	43.552	0.71146	56.39	*F*_SC_ = 0.53569 *
Within populations	73	45.016	0.61666	48.87	*F*_ST_ = 0.51128 *
**Groups A–E**					
Among groups	4	54.55	1.34029	73.89	*F*_CT_ = 0.73889 *
Among populations within groups	11	5.226	0.00054	0.03	*F*_SC_ = 0.00115 *
Within populations	48	22.708	0.47309	26.08	*F*_ST_ = 0.73919 *

d.f., degree of freedom; Ss, Sum of squares; Vc, Variance components; %Va, Percentage of variation; Fi, Fixation index; groups A–E classified based on SAMOVA (see Figure 3); * *p*-value < 0.01.

## Data Availability

All data are available upon request.

## Supplementary Materials

The following supporting information can be downloaded at: https://www.mdpi.com/article/10.3390/ani15203004/s1, Table S1: Nucleotide Materials: The following supporting and amino acid sequence variation in *RAG1* gene of *Gekko gecko*. Table S2: Statistically proposed grouping by Spatial Analysis of Molecular Variance (SAMOVA).

## References

[1] Fieldsend T.W., Rösler H., Krysko K.L., Harman M.E.A., Mahony S., Collins T.M. (2025). Genotypic and Phenotypic Evidence Reveals the Introduction of Two Distinct Forms of the Non-Native Reptile *Gekko gecko* to Southern Florida. Asain Herpetol. J..

[2] Bauer A. (2009). Geckos in Traditional Medicine: Forensic Implications. Appl. Herpetol..

[3] Nijman V., Todd M., Shepherd C.R., Gower D.J., Johnson K.G., Richardson J.E., Rosen B.R., Williams S.T. (2012). Wildlife Trade as an Impediment Wildlife trade as an impediment to conservation as exemplified by the trade in reptiles in Southeast Asia. Biotic Evolution and Environmental Change in Southeast Asia.

[4] Laoong S., Sribundit W. (2006). Diet of Tokay Gecko (*Gekko gecko*) in Eastern and Northern Regions of Thailand. Wildl. Yearb..

[5] Kongbuntad W., Tantrawatpan C., Pilap W., Jongsomchai K., Chanaboon T., Laotongsan P., Petney T.N., Saijuntha W. (2016). Genetic Diversity of the Red-Spotted Tokay Gecko (*Gekko gecko* Linnaeus, 1758) (Squamata: Gekkonidae) in Southeast Asia Determined with Multilocus Enzyme Electrophoresis. J. Asia-Pac. Biodivers..

[6] Saijuntha W., Sedlak S., Agatsuma T., Jongsomchai K., Pilap W., Kongbuntad W., Tawong W., Suksavate W., Petney T.N., Tantrawatpan C. (2019). Genetic Structure of the Red-Spotted Tokay Gecko, *Gekko gecko* (Linnaeus, 1758) (Squamata: Gekkonidae) from Mainland Southeast Asia. Asian Herpetol. Res..

[7] Caillabet O. (2013). The Trade in Tokay Geckos in South-East Asia: A Case Study on Medicinal Claims in Peninsular Malaysia.

[8] Qin X.-M., Li H.-M., Zeng Z.-H., Zeng D.-L., Guan Q.-X. (2012). Genetic Variation and Differentiation of *Gekko gecko* from Different Populations Based on Mitochondrial Cytochrome *b* Gene Sequences and Karyotypes. Zool. Sci..

[9] Zhang Y.Y., Mo X.C., Zeng W.M., Hu D.N. (2006). A Molecular Phylogeny of Red Tokay and Black Tokay (*Gekko gecko*) Based on Mitochondrial 12S rRNA Gene Sequences. Guangxi Med. J..

[10] Yu X., Peng Y., Aowphol A., Ding L., Brauth S.E., Tang Y.-Z. (2011). Geographic Variation in the Advertisement Calls of *Gekko gecko* in Relation to Variations in Morphological Features: Implications for Regional Population Differentiation. Ethol. Ecol. Evol..

[11] Qin X., Liang Y., Huang X. (2005). RAPD Analysis on Genetic Divergence and Phylogenesis of *Gekko gecko* from Different Areas. Chin. J. Zool..

[12] Wang G., Gong S., Jiang L., Peng R., Shan X., Zou D., Yang C., Zou F. (2013). Genetic Variability of the Tokay Gecko Based on Mitochondrial and Nuclear DNA. Mitochondrial DNA.

[13] Fieldsend T.W., Krysko K.L., Sharp P., Collins T.M. (2021). Provenance and Genetic Diversity of the Non-Native Geckos *Phelsuma grandis* Gray 1870 and *Gekko Gecko* (Linnaeus 1758) in Southern Florida, USA. Biol. Invasions.

[14] Shen X.-X., Liang D., Zhang P. (2012). The Development of Three Long Universal Nuclear Protein-Coding Locus Markers and Their Application to Osteichthyan Phylogenetics with Nested PCR. PLoS ONE.

[15] Bennett D. (1999). Expedition Field Techniques: Reptiles and Amphibians.

[16] Bennett R.A. (1998). Reptile Anesthesia. Semin. Avian Exot. Pet. Med..

[17] Larkin M.A., Blackshields G., Brown N.P., Chenna R., McGettigan P.A., McWilliam H., Valentin F., Wallace I.M., Wilm A., Lopez R. (2007). Clustal W and Clustal X Version 2.0. Bioinformatics.

[18] Hall T.A. (1999). BioEdit: A User-Friendly Biological Sequence Alignment Editor and Analysis Program for Windows 95/98/NT. Nucleic Acids Symp. Ser..

[19] Librado P., Rozas J. (2009). DnaSP v5: A Software for Comprehensive Analysis of DNA Polymorphism Data. Bioinformatics.

[20] Kimura M. (1980). A Simple Method for Estimating Evolutionary Rates of Base Substitutions through Comparative Studies of Nucleotide Sequences. J. Mol. Evol..

[21] Tamura K., Stecher G., Kumar S. (2021). MEGA11: Molecular Evolutionary Genetics Analysis Version 11. Mol. Biol. Evol..

[22] Leigh J.W., Bryant D. (2015). PopART: Full-feature software for haplotype network construction. Methods Ecol. Evol..

[23] Excoffier L., Lischer H.E.L. (2010). Arlequin Suite Ver 3.5: A New Series of Programs to Perform Population Genetics Analyses under Linux and Windows. Mol. Ecol. Resour..

[24] Dupanloup I., Schneider S., Excoffier L. (2002). A simulated annealing approach to define the genetic structure of populations. Mol. Ecol..

[25] Nylander J.A.A. (2004). MrModeltest v2. Program Distributed by the Author. Evolutionary Biology Centre, Uppsala University. Ampignons de l’Équateur (Pugillus IV). Bull. L’herbier Boissier.

[26] Mesquite Project. https://www.mesquiteproject.org/.

[27] Ronquist F., Teslenko M., Van Der Mark P., Ayres D.L., Darling A., Höhna S., Larget B., Liu L., Suchard M.A., Huelsenbeck J.P. (2012). MrBayes 3.2: Efficient Bayesian Phylogenetic Inference and Model Choice Across a Large Model Space. Syst. Biol..

[28] Rambaut A., Drummond A.J., Xie D., Baele G., Suchard M.A. (2018). Posterior Summarization in Bayesian Phylogenetics Using Tracer 1.7. Syst. Biol..

[29] Puillandre N., Lambert A., Brouillet S., Achaz G. (2012). ABGD, Automatic Barcode Gap Discovery for primary species delimitation. Mol. Ecol..

[30] Puillandre N., Brouillet S., Achaz G. (2021). ASAP: Assemble species by automatic partitioning. Mol. Ecol. Resour..

[31] Zhang J., Kapli P., Pavlidis P., Stamatakis A. (2013). A General Species Delimitation Method with Applications to Phylogenetic Placements. Bioinformatics.

[32] Vences M., Miralles A., Brouillet S., Ducasse J., Fedosov A., Kharchev V., Kumari S., Patmanidis S., Puillandre N., Scherz M.D. (2021). iTaxoTools 0.1: Kickstarting a specimen-based software toolkit for taxonomists. Megataxa.

[33] Zhang Y., Chen C., Li L., Zhao C., Chen W., Huang Y. (2014). Insights from ecological niche modeling on the taxonomic status of the black- and red-spotted tokay (*Gekko gecko*). Ecol. Evol..

[34] Brock K.M., McTavish E.J., Edwards D.L. (2021). Color Polymorphism is a Driver of Diversification in the Lizard Family Lacertidae. Syst. Biol..

